# Evaluating Psychophysical Polarity Sensitivity as an Indirect Estimate of Neural Status in Cochlear Implant Listeners

**DOI:** 10.1007/s10162-019-00718-2

**Published:** 2019-04-04

**Authors:** Kelly N. Jahn, Julie G. Arenberg

**Affiliations:** 1 Department of Speech and Hearing Sciences, University of Washington, 1417 NE 42nd St., Seattle, WA 98105 USA; 2 Massachusetts Eye and Ear, 243 Charles St., Boston, MA 02114 USA; 3 Department of Otolaryngology, Harvard Medical School, Boston, MA 02115 USA

**Keywords:** polarity effect, electrode position, imaging, psychophysics, electrical field imaging, electrode-neuron interface

## Abstract

The physiological integrity of spiral ganglion neurons is presumed to influence cochlear implant (CI) outcomes, but it is difficult to measure neural health in CI listeners. Modeling data suggest that, when peripheral processes have degenerated, anodic stimulation may be a more effective neural stimulus than cathodic stimulation. The primary goal of the present study was to evaluate the emerging theory that polarity sensitivity reflects neural health in CI listeners. An ideal in vivo estimate of neural integrity should vary independently of other factors known to influence the CI electrode-neuron interface, such as electrode position and tissue impedances. Thus, the present analyses quantified the relationships between polarity sensitivity and (1) electrode position estimated via computed tomography imaging, (2) intracochlear resistance estimated via electrical field imaging, and (3) focused (steered quadrupolar) behavioral thresholds, which are believed to reflect a combination of local neural health, electrode position, and intracochlear resistance. Eleven adults with Advanced Bionics devices participated. To estimate polarity sensitivity, electrode-specific behavioral thresholds in response to monopolar, triphasic pulses where the central high-amplitude phase was either anodic (CAC) or cathodic (ACA) were measured. The polarity effect was defined as the difference in threshold response to the ACA compared to the CAC stimulus. Results indicated that the polarity effect was not related to electrode-to-modiolus distance, electrode scalar location, or intracochlear resistance. Large, positive polarity effects, which may indicate SGN degeneration, were associated with relatively high focused behavioral thresholds. The polarity effect explained a significant portion of the variation in focused thresholds, even after controlling for electrode position and intracochlear resistance. Overall, these results provide support for the theory that the polarity effect may reflect neural integrity in CI listeners. Evidence from this study supports further investigation into the use of polarity sensitivity for optimizing individual CI programming parameters.

## INTRODUCTION

Cochlear implants (CIs) stimulate the auditory system by directly depolarizing spiral ganglion neurons (SGNs), and the physiological integrity of the SGNs may contribute to a patient’s success with a CI. In fact, variability in phoneme, word, and sentence perception scores across CI listeners is partially explained by demographic variables that are implicitly related to neural health, such as duration of deafness, age, and hearing loss etiology (Friedland et al. 2010; Lazard et al. 2012; Holden et al. 2013). In post-mortem temporal bone studies, total SGN counts decrease with increasing age and duration of deafness and vary as a function of etiology (Nadol et al. 1989, Nadol 1997). Moreover, some investigations have shown that in vivo estimates of SGN density relate to speech perception performance in CI listeners (e.g., Kim et al. 2010; Zhou and Pfingst 2014; DeVries et al. 2016; Scheperle 2017; Schvartz-Leyzac and Pfingst 2018). While SGN density constitutes an important aspect of neural health, the status of the peripheral processes may also contribute to CI outcomes. However, less is understood about how to quantify peripheral process integrity in vivo.

Recent evidence suggests that sensitivity to stimulus polarity may provide insight into the health of the peripheral processes. The polarity effect represents the difference in psychophysical or electrophysiological responses to positive (anodic) and negative (cathodic) electrical current. Biophysical modeling data show that the site of spike initiation differs for anodic and cathodic polarities (Rattay et al. 2001a, b; Joshi et al. 2017; Resnick et al. 2018). Specifically, anodic current is more effective at exciting the central axon, whereas cathodic current is more effective at exciting the peripheral processes. When the peripheral processes have degenerated, anodic stimulation generates action potentials at lower current levels than cathodic stimulation (Rattay et al. 2001a, b; Joshi et al. 2017; Resnick et al. 2018). If this theory holds true in CI listeners, then large, positive polarity effects may suggest degeneration of adjacent SGNs.

In CI listeners, better sensitivity to anodic than to cathodic stimulation is consistently observed at suprathreshold levels, which may reflect a proximal shift in spike initiation at high current levels (Macherey et al. 2006, 2008; van Wieringen et al. 2008; Undurraga et al. 2010, 2013; Macherey et al. 2017; Hughes et al. 2017, 2018). Polarity sensitivity at low current levels, however, may provide more insight into the variation in local neural status than suprathreshold measurements. In fact, recent evidence suggests that the polarity effect at threshold varies both across- and within-ears (Macherey et al. 2017; Carlyon et al. 2018). Although neural health also varies across the electrode array, so do non-physiological factors such as electrode position relative to the SGNs and intracochlear bone and tissue growth (reviewed by Bierer 2010).

An ideal in vivo estimate of neural status would vary independently of electrode placement and tissue impedances. However, it is unknown whether polarity effect measurements are influenced by electrode position or intracochlear resistance in CI listeners. The primary goal of the present study was to evaluate the theory that polarity sensitivity reflects neural status by quantifying its relationship with electrode-to-modiolus distance, electrode scalar location, and intracochlear resistance. Because computational studies have used highly simplified models to describe polarity sensitivity, there was no a priori expectation of whether the polarity effect would correlate with electrode position or intracochlear resistance.

A secondary goal was to evaluate the relationship between the polarity effect and behavioral thresholds measured in response to a spatially focused electrode configuration (i.e., focused thresholds). Focused threshold levels are believed to reflect the cumulative contributions of local electrode placement, bone and tissue growth, and neural health (Bierer 2010). Specifically, channels with relatively high focused thresholds are often located farther from target neurons (Long et al. 2014; DeVries et al. 2016; DeVries and Arenberg 2018a) and have lower intracochlear resistance values (Bierer et al. 2015b) and smaller evoked potential amplitudes (DeVries et al. 2016) than channels with lower focused thresholds. In line with the theory that polarity sensitivity reflects neural health, we hypothesized that the polarity effect would explain a significant portion of the variation in focused thresholds, even after controlling for the contributions of electrode position and intracochlear resistance. The results of this study will provide evidence either in support of or against the use of polarity sensitivity as an estimate of neural status in CI listeners. Ultimately, in vivo estimates of peripheral process integrity may improve our understanding of how aspects of neural health other than SGN density relate to CI performance, and whether that information can be used to optimize device programming.

## METHODS

### Subjects

Eleven adults (6 males) who were unilaterally implanted with Advanced Bionics HiRes90K devices participated (Table 1). Subjects ranged in age from 27 to 87 years (*M* = 62.00 years, *SD* = 18.32). Two subjects (S49 and S53) were pre-lingually deafened (diagnosed with severe to profound sensorineural hearing loss before the age of 4 years) and one (S40) was peri-lingually deafened (diagnosed with severe to profound sensorineural hearing loss at age 4). The remaining eight subjects became deaf in adulthood. All subjects were fluent English speakers and used spoken language to communicate. One subject (S54) learned English as a second language. Each subject provided written informed consent and all procedures were approved by the University of Washington Human Subjects Division.

**Table 1:** Demographic information for all 11 subjects including ear implanted, chronological age, age diagnosed with a profound hearing loss, age at implantation, duration of deafness, etiology (if known), and electrode array type

ID	Ear	Age (years)	Age at profound HL (years)	Age implanted (years)	Duration of deafness (years)	Etiology	Electrode array
S22	R	78	55	66	11	Suspected genetic	1-J Helix
S29	L	87	46	76	30	Noise exposure	HiFocus 1J
S40	L	56	4	50	46	EVA	HiFocus 1J
S43	R	72	49	67	18	Noise exposure	Mid-scala
S46	R	69	40	64	24	Suspected genetic	HiFocus 1J
S47	R	40	26	36	10	Unknown	Mid-scala
S49	R	45	1.5	44	42.5	Genetic	Mid-scala
S50	R	76	18	71	53	Unknown	HiFocus 1J
S52	R	71	59	65	6	Unknown	HiFocus 1J
S53	R	56	1	44	43	Meningitis	1-J Helix
S54	L	27	7	23	16	EVA	Mid-scala
Mean (*SD*)		62.0 (*18.3*)	27.9 (*22.8*)	55.5 (*16.8*)	27.6 (*16.3*)		

*EVA*, enlarged vestibular aqueduct

### Computed Tomography Imaging

Electrode position was estimated using computed tomography (CT) imaging. Subjects enrolled in a series of CT-based studies in our laboratory between the years 2013 and 2016. CT scans were obtained at the time of enrollment. Imaging was not repeated, as substantial electrode migration is rare (Rader et al. 2016; Dietz et al. 2016), and no subjects demonstrated clinical signs of array migration over the course of the experiments; that is, electrode impedances, behavioral thresholds, and speech perception scores were stable over time (Rader et al. 2016). Thus, the risks of obtaining serial CT scans (e.g., exposure to radiation, financial costs) outweighed the benefits. All other psychophysical and objective measures were obtained simultaneously at the time of each experiment. Nine of the 11 subjects participated in more than one experiment in our laboratory that incorporated these CT images (DeVries et al. 2016; DeVries and Arenberg 2018a). Subjects S50 and S52 were not included in the prior publications.

**Fig. 1:**
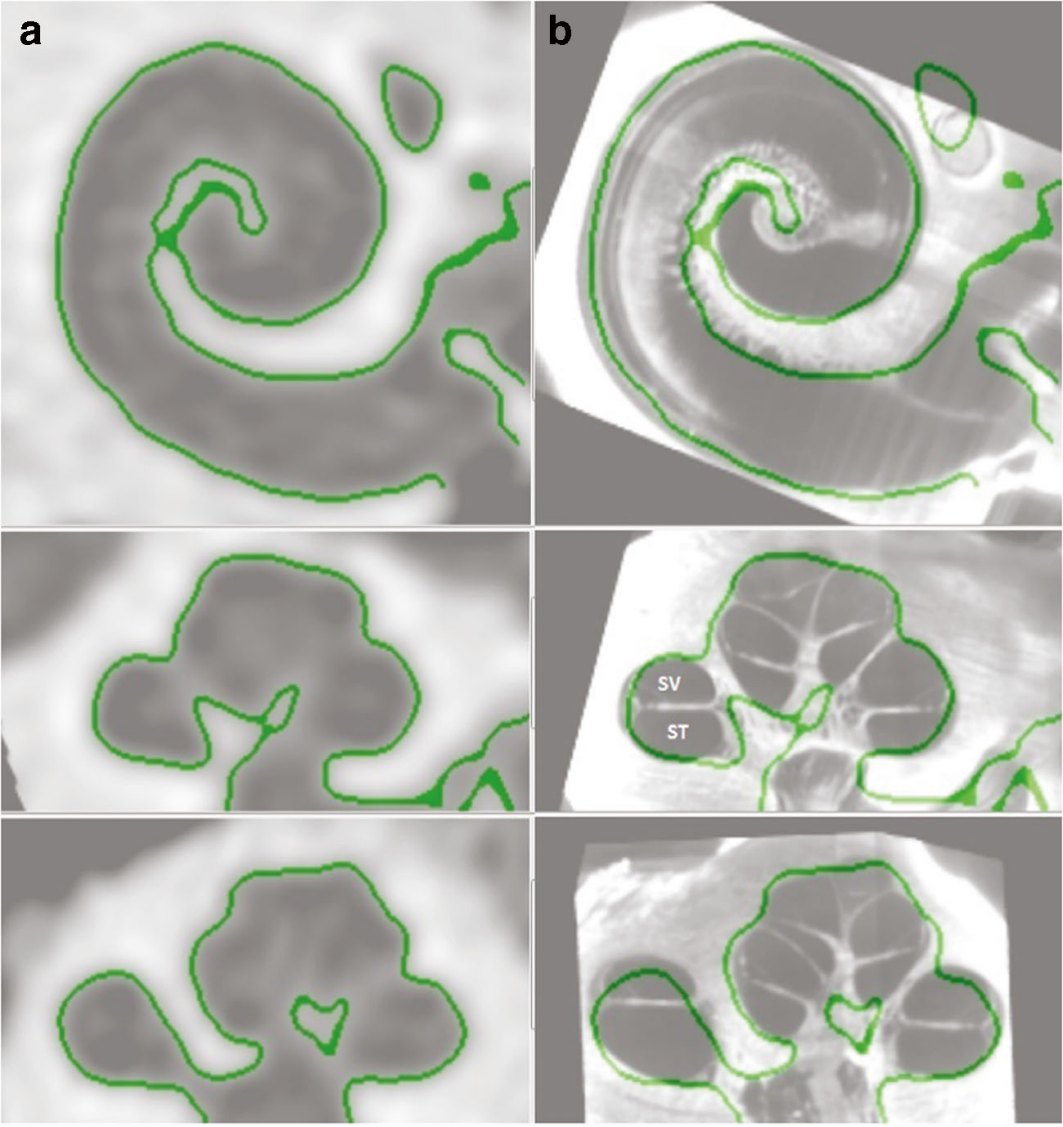
Cochlear wall as defined by the computed tomography (CT) scan (column **a**) and an atlas selected from 10 micro CTs and 1 OPFOS (orthogonal-plane fluorescence optical sectioning) that was aligned by rigid registration to create best fit (column **b**). SV denotes the estimated location of scala vestibuli and ST denotes the estimated location of scala tympani

**Fig. 2:**
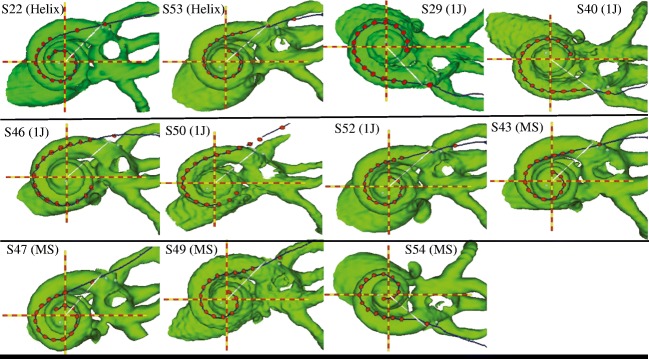
3D cochlear reconstructions from all 11 subjects, arranged in order of electrode array type (Helix followed by 1J followed by mid-scala). MS represents the mid-scala array. Red dots represent the electrodes, with the outermost dot representing the insertion depth marker. The red and yellow lines represent the X and Y axes that are used to determine the insertion angle from the white 0° insertion line

CT scans of the implanted and non-implanted ears of each subject were performed at the University of Washington Medical Center in Seattle, Washington. The scans were analyzed at Washington University in St. Louis, Missouri, using a technique developed by Skinner et al. (2007) and validated by Teymouri et al. (2011). Images of the non-implanted ear were obtained because pre-operative CT scans were not available, and because metal artifact contamination in the implanted ear interferes with the ability to accurately identify individual electrodes and cochlear anatomy. Using ANALYZE software (Mayo Clinic, Rochester; Robb 2001), the image of the non-implanted ear was co-registered with the image of the implanted ear to identify structural anatomy and to optimize resolution of the electrodes. To further improve visualization of the scalar position of the electrode array and the individual electrodes, each of the 11 cochlear atlases was aligned, through translation, rotation, and scaling, to find a best fit to the cochlear wall of the composite CT (Fig. 1). The atlases assist in the localization of non-bony cochlear structures and were derived from 10 Micro CTs and 1 orthogonal-plane fluorescence optical sectioning (OPFOS) (Voie et al. 1993) obtained from cadaveric temporal bones. The composite CT images from all 11 ears are shown in Fig. 2 (see “Results” section for descriptive analyses of electrode position).

Two CT-estimated metrics of electrode position were used in the present study: electrode-to-modiolus distance and scalar location. Electrode-to-modiolus distance was calculated as the lateral distance, in millimeters (mm), of an electrode from the inner wall of the cochlea. Scalar location refers to the location of each electrode within the cochlear compartments: scala tympani (ST), intermediate, or scala vestibuli (SV). Given the relatively small cross-sectional area of the scala media, the intermediate position refers to those electrodes that approximate the position of the basilar membrane and that could not be definitively localized to either the ST or the SV.

### Electrical Stimulation

All electrical stimuli were presented and controlled using the Bionic Ear Data Collection System (BEDCS) version 1.18.315 (Advanced Bionics, Valencia, CA). Custom MATLAB scripts controlled the BEDCS software (MathWorks, Inc. Natick, MA). Stimuli were verified using a reference implant and digital storage oscilloscope.

### Polarity Effect

Polarity sensitivity was assessed on each available channel in each subject. This was accomplished by measuring single-channel behavioral thresholds in response to each of two triphasic pulses, in which the high-amplitude central phase was either anodic (CAC) or cathodic (ACA). Several investigations have demonstrated that asymmetric pulse shapes (e.g., pseudomonophasic, triphasic, or quadruphasic) are more effective than symmetric biphasic pulses for studying polarity sensitivity in human behavioral experiments (Macherey et al. 2006, 2008; Carlyon et al. 2013; Macherey et al. 2017). Recently, Carlyon et al. (2018) studied psychophysical polarity sensitivity at threshold using triphasic pulses in which the central high-amplitude phase of each pulse was twice the amplitude of the first and third phases. This type of asymmetric pulse shape concentrates the charge of the polarity of interest into a short time window, while maintaining the necessary charge balance. The stimuli used in the present study were identical to those used by Carlyon et al. (2018). Stimuli were 99 pulse-per-second (pps) trains presented in a monopolar stimulation mode (43 μs/phase, 0-μs interphase gap, 400 ms duration).

The polarity effect was defined as the difference between ACA and CAC thresholds on each channel (polarity effect, in dB = ACA minus CAC). A positive polarity effect indicated that behavioral thresholds in response to the ACA stimulus were higher (i.e., worse) than those in response to the CAC stimulus. A negative polarity effect indicated the inverse.

Prior to threshold testing, most comfortable listening levels (MCLs) were obtained on each channel, in response to each polarity. This was done to avoid presenting uncomfortable stimulation levels, and to determine an appropriate starting level for threshold measurements. To measure MCL, the experimenter gradually increased the current level from a subthreshold level of 50 microamps (μA) up until the subject reported a loudness rating of “6,” corresponding to “most comfortable” on the Advanced Bionics clinical loudness scale (Advanced Bionics, Valencia, CA). The MCL ratings served as the maximum stimulus levels for the threshold procedures.

Single-channel signal detection thresholds were measured for each polarity on each available channel using an adaptive one-up/one-down staircase tracking procedure. Two adaptive tracks were completed for each polarity on each electrode, and the two values were averaged together to arrive at the final threshold estimation. If the thresholds estimated on the first two runs differed by 1 decibel (dB) or more, a third and fourth run were completed. In that case, the thresholds from each of the four runs were averaged together. The testing order of channels and polarities was randomized for each subject.

For each adaptive track, the initial presentation level was set to 90 % of MCL. For subsequent tracks, the initial presentation level was set to 50 to 98 % of MCL. On electrodes with relatively large dynamic ranges, a lower percentage of MCL was used as the starting level to reduce the number of steps necessary to estimate threshold. Conversely, a higher starting level was selected for electrodes with small dynamic ranges to ensure that the subject could adequately hear the stimulus before reaching the first reversal.

The subject indicated when he or she heard a sound by pressing the spacebar on a standard computer keyboard. If the subject responded within 3 s after stimulus presentation, the presentation level decreased. If the subject did not respond within 3 s, the level increased. The initial step size was 0.5 dB. After the first reversal, the step size reduced to 0.2 dB. Random delays ranging from 0.1 to 0.6 s were implemented prior to each stimulus presentation. The adaptive procedure terminated after eight reversals, and threshold level was estimated as the average of the last six reversals.

### Focused Behavioral Thresholds

Single-channel focused behavioral thresholds were estimated for channels 2–15 using a sweep procedure (based on Sek et al. 2005; Bierer et al. 2015a). Stimuli were biphasic, cathodic-leading pulse trains (102 μs/phase, 0-μs interphase gap, 200.4 ms duration, 997.9 pps) presented in a steered quadrupolar (sQP) stimulation mode with a current focusing coefficient of 0.9. Across studies, focused thresholds measured with cathodic-leading biphasic pulses reflect variation in CT-estimated electrode position (Long et al. 2014; DeVries et al. 2016; DeVries and Arenberg 2018a), EFI-estimated intracochlear resistance (Bierer et al. 2015b), and evoked potential estimates of SGN density (Bierer et al. 2011; DeVries et al. 2016). We thus maintained a pulse shape consistent with prior investigations (biphasic, cathodic-leading).

In sQP stimulation, a channel is comprised of four adjacent intracochlear electrodes. Two middle electrodes serve as active electrodes, whereas two outer electrodes serve as return electrodes. The current focusing coefficient, sigma (σ), specifies the fraction of current delivered through the return electrodes. Higher sigma values indicate greater current focusing, such that σ = 1 represents the highest possible degree of current focusing. In the present study, a highly focused coefficient of σ = 0.9 was selected to capture local variability in the ENI while maintaining perceptible current levels that were below voltage compliance limits (Bierer 2007). Note that Advanced Bionics devices have 16 intracochlear electrodes; however, sQP thresholds cannot be measured on channels 1 and 16 due to the need for two intracochlear return electrodes.

A fast threshold measurement procedure based on a variation of the Bekesy tracking technique was used to obtain sQP thresholds across the electrode array (Bierer et al. 2015a). Current was steered between the two active electrodes by changing the steering coefficient, alpha (α). When α = 0, all current was delivered through the more apical of the two active electrodes; when α = 1, all current was steered through the most basal active electrode. By convention, an electrode channel number is equivalent to the basal active electrode when α = 1. This convention can be maintained for electrodes 3 to 15. However, because the sQP configuration requires four electrodes, it is not possible to steer current for electrode 2 in the same manner as for electrodes 3–15. Instead, the same set of electrodes must be used for both channel 2 and channel 3, but an *α* value of 0 is specified to center the current on electrode 2. This arrangement is referred to as “channel 2.”

Prior to initiating the threshold sweep procedure, MCLs were measured for the sQP stimuli across channels 2–15 using the Advanced Bionics loudness rating scale and the same procedure described in the previous section. MCL was then set as the upper limit of stimulation for the threshold measurements. To measure threshold, pulse trains were presented starting at 6 dB below MCL and swept across the electrode array with alpha increasing from 0 to 1 in steps of 0.1. Each 200.4 ms pulse train was followed by a 300 ms silent interval, resulting in a repetition rate of approximately 500 ms. The alpha value changed every 1000 ms, such that two successive presentations of the same electrode and alpha combination occurred during each sweep. This process repeated uninterrupted for each successive set of electrodes until all available sets (active electrodes 2–15) had been tested, constituting a single run.

During the sweep, the listener was instructed to continuously depress the spacebar when he or she could perceive a sound, and to release the spacebar when he or she could no longer perceive a sound. The current changed in step sizes of 1 dB. When the spacebar was depressed, the current level decreased by 1 dB on each stimulus presentation. Conversely, when the spacebar was released, the current level increased by 1 dB on each stimulus presentation. Two successive pulse trains were presented for each alpha value. Because the task is based on Bekesy tracking principles, both the alpha value and the current level change while the spacebar is depressed in order to continuously sweep the stimulus across the electrode array. The reader is referred to Fig. 2 of Bierer et al. (2015a) for a visual representation of this procedure.

Participants completed two forward runs, sweeping from channels 2 to 15 (apical to basal), and two reverse runs, sweeping from channels 15 to 2 (basal to apical). Final threshold estimates were obtained by calculating a weighted average of consecutive current levels along the forward and reverse sweeps at integer channel numbers (as in Bierer et al. 2015a). Thresholds were calculated at integer channel numbers for direct comparison to the other measures in this study. This current steering method also provides threshold data in 0.1 alpha increments in-between the integer channel numbers; however, despite the extra data collection, thresholds across the electrode array are still obtained up to four times faster with this procedure compared to traditional two-alternative forced-choice methods (Bierer et al. 2015a).

### Electrical Field Imaging

Stimuli were single biphasic, anodic-leading pulses (100 μs duration, either 50 or 100 μA in amplitude) presented in a monopolar stimulation mode at a rate of 16.6 per second. EFI uses low-level pulses to estimate the distribution of electrical current in the cochlea. Note that the leading polarity of the EFI stimulus does not affect the voltage measures, and that the low-level stimuli were sub-threshold for all subjects in this study. Ten pulses were presented on each electrode consecutively, progressing from electrode 1 to electrode 16 (apical to basal). While the pulses were presented, voltage was recorded on every electrode at a 56 kHz sampling rate. Data analysis was performed offline with a custom MATLAB program. The analysis was modeled after that of Vanpoucke et al. (2004). Briefly, the voltage that was measured at each recording electrode in response to each of the ten pulses was averaged. Voltage was then scaled to resistance units (Ohms) by dividing by the applied current. Signal amplitude at each recording electrode was calculated as half the difference between the positive and negative voltage excursions.

The 16 × 16 impedance matrix was transformed to solve a lumped parameter resistor network. The solution yielded 16 transversal resistances (R_trans_), 15 longitudinal resistances (R_long_), and 16 total resistances (R_total_). R_long_ and R_trans_ values were estimated using least squares optimization and a localized weighting scheme to improve the EFI profile fit. R_long_ represents localized current flow along the length of the cochlea, whereas R_trans_ includes resistance pathways through the osseous spiral lamina. R_total_ was calculated as the peak of the reconstructed EFI profile, which was based on the solution to the ladder network. A detailed explanation of these analyses is discussed by Vanpoucke et al. (2004). All three resistance measurements are highly correlated with one another, so only R_long_ was used in the present analyses. R_long_ was selected because it has been hypothesized that the majority of current from an electrode flows longitudinally along the cochlear duct (Jolly et al. 1996; Briare and Frijns 2000).

### Statistical Analyses

Data were analyzed using R Version 3.3.1 (R Core Team 2016). To account for repeated measurements within the same subjects, either repeated measures correlations (Bakdash and Marusich 2017) or linear mixed-effects models were used for all analyses that involved electrode-specific data. In such cases, subjects were included in the models as random factors. To minimize small sample bias in estimation, all linear mixed-effects models were fit using restricted maximum likelihood (REML) parameter estimates (McNeish 2017). Repeated measures analyses were performed using the lme4 (Bates et al. 2015), lmerTest (Kuznetsova et al. 2017), MuMIn (Bartón 2018), and rmcorr (Bakdash and Marusich 2018) packages in R.

## RESULTS

### Comparisons of Polarity Effect, Electrode Position, and Intracochlear Resistance

Figure 2 shows the 3D cochlear reconstructions of all subjects, arranged in order of electrode array type. Two subjects had a 1J-Helix array (S22 and S53), five subjects had a 1J array (S29, S40, S46, S50, and S52), and four subjects had a mid-scala array (S43, S47, S49, and S54). Each of the three types of electrode arrays is designed to achieve a different position within the cochlea (Dhanasingh and Jolly 2017). The standard 1J array has a lateral wall design. The 1J-Helix and the mid-scala arrays are both pre-curved. The 1J-Helix should achieve a more medial position relative to the 1J, whereas the mid-scala is designed for placement in the middle of the ST. Of the 176 total electrodes in the sample, 104 electrodes (59.1 %) were located in ST, 62 electrodes (35.2 %) were located in the intermediate position, and 8 electrodes (5.5 %) were located in SV. Two of subject S50’s electrodes were extracochlear (electrodes 15 and 16). Across electrodes, electrode-to-modiolus distance ranged from 0.18 to 2.2 mm (*M* = 1.14 mm, *SD* = 0.52; see Table 2).

**Table 2:** Means and standard deviations for sQP threshold, polarity effect, electrode-to-modiolus distance (EMD), and intracochlear resistance. Data are averaged across electrodes 2–15

ID	sQP threshold (dB re: 1 μA)	Polarity effect (dB)	EMD (mm)	Resistance (Ohms)
S22	44.30 (*5.90*)	− 0.45 (*2.08*)	1.14 (*0.35*)	208.03 (*62.63*)
S29	47.76 (*3.00*)	0.61 (*0.57*)	1.50 (*0.24*)	333.75 (*90.58*)
S40	55.76 (*1.37*)	2.29 (*0.59*)	1.80 (*0.22*)	595.44 (*607.13*)
S43	43.73 (*3.68*)	− 0.06 (*1.06*)	0.88 (*0.45*)	274.58 (*68.53*)
S46	51.69 (*1.53*)	0.08 (*0.57*)	1.79 (*0.34*)	583.70 (*186.62*)
S47	35.29 (*4.04*)	0.66 (*0.97*)	0.93 (*0.46*)	237.57 (*135.58*)
S49	50.71 (*2.15*)	− 0.03 (*1.36*)	0.94 (*0.47*)	1122.99 (*642.40*)
S50	45.82 (*5.08*)	1.05 (*1.83*)	1.37 (*0.27*)	567.82 (*646.46*)
S52	39.01 (*1.92*)	− 0.48 (*1.03*)	0.71 (*0.30*)	345.00 (*185.68*)
S53	45.55 (*2.62*)	0.34 (*1.20*)	0.66 (*0.17*)	274.57 (*127.61*)
S54	47.14 (*5.77*)	0.09 (*1.55*)	0.89 (*0.46*)	1064.25 (*1002.10*)
Mean (*SD*)	46.01 (*6.51*)	0.36 (*1.42*)	1.14 (>*0.52*)	496.95 (*518.07*)

**Fig. 3:**
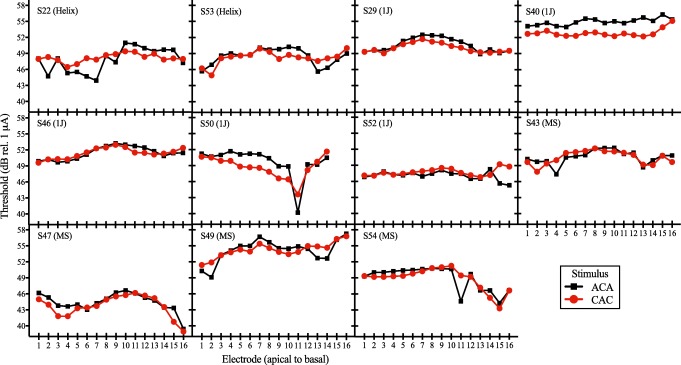
Individual subjects’ electrode-specific thresholds in response to each of the two triphasic monopolar stimuli. ACA refers to the stimulus with a cathodic central phase and CAC refers to the stimulus with an anodic central phase. Thresholds in response to the ACA stimulus are depicted by black squares. Thresholds in response to the CAC stimulus are depicted by red circles. Threshold (in dB relative to 1 μA) is shown on the ordinate and electrode number (ordered from apical to basal) is shown on the abscissa. Subjects are arranged in order of electrode array type, with the electrode array displayed in parentheses. MS represents the mid-scala array. The order of subjects is the same as in Fig. 3

Figure 3 shows individual site-specific thresholds in response to the ACA and CAC stimuli. Black squares indicate responses to the ACA stimulus, whereas red circles indicate responses to the CAC stimulus. Higher (i.e., worse) thresholds in response to the ACA stimulus compared to the CAC stimulus indicate a positive polarity effect. Conversely, higher (i.e., worse) thresholds in response to the CAC stimulus compared to the ACA stimulus indicate a negative polarity effect. The polarity effect could not be measured on electrodes 15 and 16 for subject S50, because those electrodes were extracochlear and stimulation did not result in auditory percepts.

Across electrodes, the polarity effect ranged from − 4.83 to 3.54 dB (*M* = 0.36 dB, *SD* = 1.42; see Table 2). The polarity effect varied both across subjects and across the electrode array within the same subject. For example, subject S40 shows consistently large, positive polarity effects at most electrode sites (Fig. 3). Conversely, subject S22 tends to have negative polarity effects in the apical portion of the electrode array, and positive polarity effects in the basal portion of the electrode array (Fig. 3).

Intracochlear resistance also varied across- and within-subjects, ranging from 91.71 to 2718.57 Ohms (*M* = 496.95 Ohms, *SD* = 518.17; see Table 2). Prior to data analysis, histograms were plotted to determine whether the data were normally distributed. This analysis revealed that R_long_ values were highly skewed. To reduce skew, the R_long_ data were log-transformed prior to data analysis and for visualization purposes. Of note, the size of the electrode contacts differs slightly between the three electrode arrays, which may influence electrode impedances (Hughes 2012). However, R_long_ did not differ as a function of electrode array type in this study (*β* = 2.36, *F*_(2,8)_ = 1.45, *P* > 0.05).

**Fig. 4:**
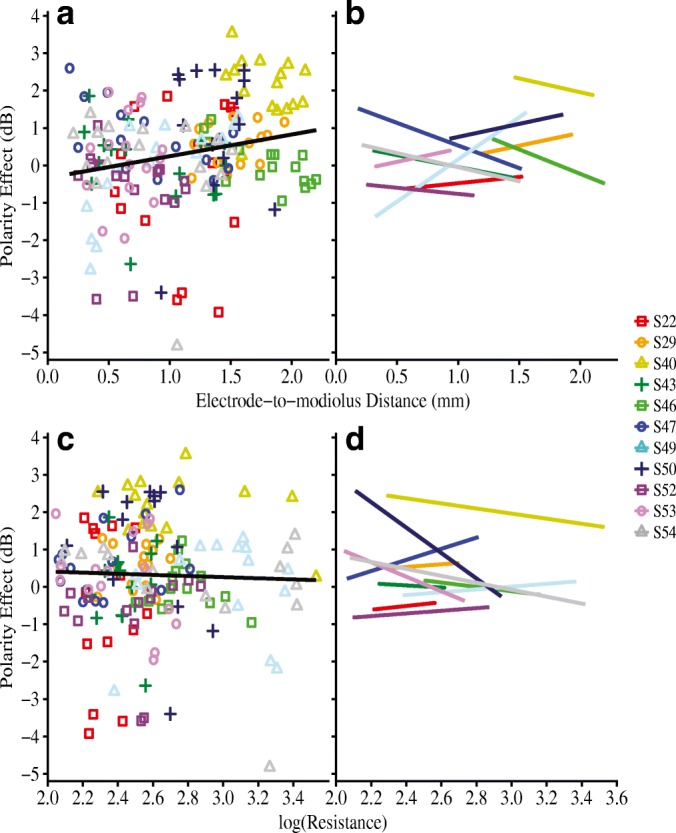
Polarity effect (in dB) as a function of **a**, **b** electrode-to-modiolus distance (in mm) and, **c**, **d** log-transformed intracochlear resistance values. Individual subjects are represented by different colors and shapes. In panels **a** and **c**, a group regression line is shown in black for ease of visualization. However, note that the black regression line does not account for non-independence of within-subject data. Panels **b** and **d** show best-fit lines for individual subjects

The first analysis assessed the relationship between the polarity effect and (1) electrode position and (2) intracochlear resistance. Figure 4 shows electrode-specific polarity effect plotted against (a) electrode-to-modiolus distance, and (c) intracochlear resistance. Individual subjects are distinguished by color and shape. The right-hand panels of Fig. 4 (b and d) show the best-fit lines for each individual. Results from a linear mixed-effects analysis showed that, across subjects, the polarity effect was not significantly predicted by electrode-to-modiolus distance (*β* = 0.11, *F*_(1,103.23)_ = 0.15, *P* = 0.70), electrode scalar location (*β* = − 0.15 for intermediate, 0.45 for SV, *F*_(2, 145.48)_ = 0.50, *P* = 0.61), or intracochlear resistance (*β* = − 0.39, *F*_(1, 106.62)_ = 0.86, *P* = 0.36). As a complement to the multilevel model, repeated measures correlations (*r*_rm_) indicated very small effect sizes for each comparison: (1) polarity effect and electrode-to-modiolus distance (*r*_rm(162)_ = − 0.02, 95 % CI [− 0.17, 0.14], *P* = 0.80) and (2) polarity effect and intracochlear resistance (*r*_rm(151)_ = − 0.13, 95 % CI [− 0.28, 0.03], *P* = 0.11). These results suggest that the polarity effect varies independently of both CT-estimated electrode position and EFI-estimated longitudinal intracochlear resistance.

### Predicting Focused Behavioral Thresholds

**Fig. 5:**
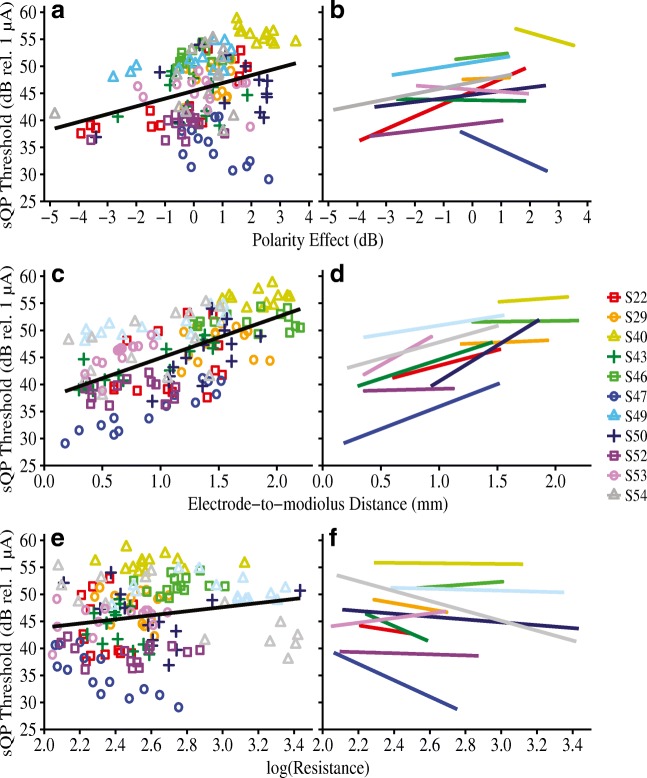
Focused (sQP) thresholds (in dB relative to 1 μA) as a function of **a**, **b** polarity effect (in dB), **c**, **d** electrode-to-modiolus distance (in mm), and **e**, **f** log-transformed intracochlear resistance values. Individual subjects are represented by different colors and shapes. In panels **a**, **c**, and **e**, a group regression line is shown in black for ease of visualization. However, note that the black regression line does not account for non-independence of within-subject data. Panels **b**, **d**, and **f** show best-fit lines for individual subjects

The second analysis assessed the relationship between the polarity effect and sQP thresholds and whether the polarity effect explains a significant portion of the variation in sQP thresholds after accounting for CT-estimated electrode position and EFI-estimated intracochlear resistance. Recall that sQP thresholds are believed to reflect the cumulative contributions of local neural health, electrode position relative to the auditory nerve, and intracochlear resistance. Figure 5 shows the relationships between electrode-specific sQP thresholds and (a) the polarity effect, (c) electrode-to-modiolus distance, and (e) intracochlear resistance. Again, individual data are distinguished by color and shape. The right-hand panels of Fig. 5 (b, d, and f) show the best-fit lines for each individual. Repeated measures correlations indicated significant relationships between focused thresholds and each of the three main predictors: (1) electrode-to-modiolus distance: *r*_rm(140)_ = 0.51, 95 % CI (0.38, 0.63), *P* < 0.001; (2) intracochlear resistance: *r*_rm(141)_ = − 0.37, 95 % CI (− 0.51, − 0.22), *P* < 0.001; and (3) the polarity effect: *r*_rm(140)_ = 0.27, 95 % CI (0.11, 0.42), *P* = 0.001. Each relationship survived Bonferroni adjustment for multiple comparisons (adjusted *α* = 0.017).

A linear mixed-effects analysis was performed to assess whether the polarity effect remained a significant predictor of sQP thresholds after controlling for electrode position and intracochlear resistance. To determine whether each variable improved the overall model fit, an Akaike information criterion with a bias correction for small sample sizes (AICc) was used for model selection (Hurvich and Tsai 1989). Statistical models with lower AICc values are more parsimonious relative to those with higher AICc. First, we specified an empty model with only subjects as random effects to predict sQP thresholds (AICc = 877.62). Next, we consecutively added each of the following fixed effects: electrode-to-modiolus distance (AICc = 824.33), scalar location (AICc = 808.72), intracochlear resistance (AICc = 803.49), and the polarity effect (AICc = 792.99). The model fit improved with the addition of each predictor, and the lowest AICc value was associated with the model that included all four predictors. So, the final model specified electrode-to-modiolus distance, scalar location, intracochlear resistance, and the polarity effect as independent variables. The dependent variable was sQP threshold.

Traditional *R*^2^ values are not valid for linear mixed-effects models. However, Nakagawa and Schielzeth (2013) propose two types of pseudo-*R*^2^ values that can provide an indication of the variability explained by a multilevel model: (1) marginal *R*^2^ (*R*^2^_marginal_), which represents the proportion of the total variance explained by the fixed effects, and (2) conditional *R*^2^ (*R*^2^_conditional_), which represents the proportion of the variance explained by both the fixed and random effects. The difference between the *R*^2^_marginal_ and *R*^2^_conditional_ reflects the variability in the random effects; here, this represents across-subject variability.

Results from the linear mixed-effects analysis (*R*^2^_marginal_ = 0.27, *R*^2^_conditional_ = 0.80) showed that the polarity effect (*β* = 0.73, *F*_(1, 138.54)_ = 14.83, *P* < 0.001), electrode-to-modiolus distance (*β* = 5.46, *F*_(1,144.60)_ = 57.36, *P* < 0.001), scalar location (*β* = − 1.18 for intermediate, 3.52 for SV, *F*_(2, 141.61)_ = 4.97, *P* = 0.01), and intracochlear resistance (*β* = − 2.42, *F*_(1, 144.99)_ = 4.27, *P* = 0.04) were all significantly predictive of sQP thresholds. After adjustment for multiple comparisons (Tukey), results showed that thresholds were higher for electrodes located in SV relative to those located in the intermediate position (*t*_(141.69)_ = 2.91, *P* = 0.01). There were no significant differences in sQP threshold between electrodes located in SV compared to ST (*P* = 0.08) or in ST compared to the intermediate position (*P* = 0.21).

Note that the group regression line in Fig. 5(e–f), which depicts the relationship between sQP thresholds and R_long_, appears to have a modest positive trajectory; however, the *β* coefficient for the R_long_ predictor is − 2.42 and the repeated measures correlation coefficient (*r*_rm_) is − 0.37. This suggests that higher sQP thresholds are associated with lower R_long_ values, demonstrating the importance of statistically accounting for clustered data.

Overall, the results indicate that, across subjects, relatively high sQP thresholds are associated with large, positive polarity effects, distant electrode position relative to the modiolus, and low intracochlear resistance. Electrodes with high sQP thresholds are also more likely to be translocated to the SV than electrodes with low sQP thresholds. Moreover, the polarity effect significantly predicts sQP thresholds, even after controlling for electrode-to-modiolus distance, electrode scalar location, and intracochlear resistance.

Despite these results, it is evident from Figs. 3, 4 and 5 that the within-subject relationships between variables are not the same for every participant. Table 3 shows within-subject correlations between sQP thresholds and electrode-to-modiolus distance (threshold-EMD), the polarity effect (threshold-PE), and R_long_ (threshold-R_long_) for each individual subject. It was noted that five out of 11 subjects (S47, S43, S53, S49, and S50) had strong, positive correlations between focused thresholds and electrode-to-modiolus distance, with correlation coefficients (*r*) ranging from 0.71 to 0.94 (Cohen 1988). Each of those five threshold-EMD correlations was statistically significant (*P*s < 0.05). This suggests that the variation in sQP thresholds for those five subjects is largely explained by variation in electrode position. The remaining six subjects had weak-to-moderate threshold-EMD correlations (*r* = 0.06 to 0.48) that were not statistically significant (*P*s > 0.05). For that group, electrode position does not account for much of the variability in sQP thresholds.

**Table 3:** Individual correlation coefficients (*r*) and *P* values for correlations between sQP thresholds (dB rel. 1 μA) and each of the following variables: electrode-to-modiolus distance (EMD; in mm), polarity effect (PE; in dB), and intracochlear resistance (R_long_; in Ohms); data are arranged in descending order by strength of threshold-EMD correlation. Significant correlations (*P* < 0.05) are italicized

	Threshold-EMD		Threshold-PE		Threshold-R_long_	
*ID*	*r*	*P*	*r*	*P*	*r*	*P*
S47	*0.94*	< *0.001*	− *0.58*	*0.03*	− *0.82*	*0.001*
S43	*0.87*	< *0.001*	− 0.02	0.94	− 0.40	0.08
S53	*0.79*	< *0.001*	− 0.17	0.55	0.09	0.24
S49	*0.74*	*0.002*	*0.54*	*0.04*	− 0.23	0.41
S50	*0.71*	*0.01*	0.24	0.43	− 0.39	0.61
S54	0.48	0.08	0.28	0.33	− *0.78*	< *0.001*
S22	0.34	0.23	*0.82*	< *0.001*	− 0.24	0.29
S40	0.23	0.44	− *0.68*	*0.01*	0.01	0.91
S52	0.09	0.77	0.34	0.24	− 0.13	0.96
S29	0.07	0.81	0.04	0.90	− 0.21	0.49
S46	0.06	0.83	0.23	0.43	0.40	0.49

Pearson correlations are shown for the threshold-PE and threshold-EMD comparisons

Non-parametric Spearman correlations are shown for the threshold-R_long_ comparisons

**Fig. 6:**
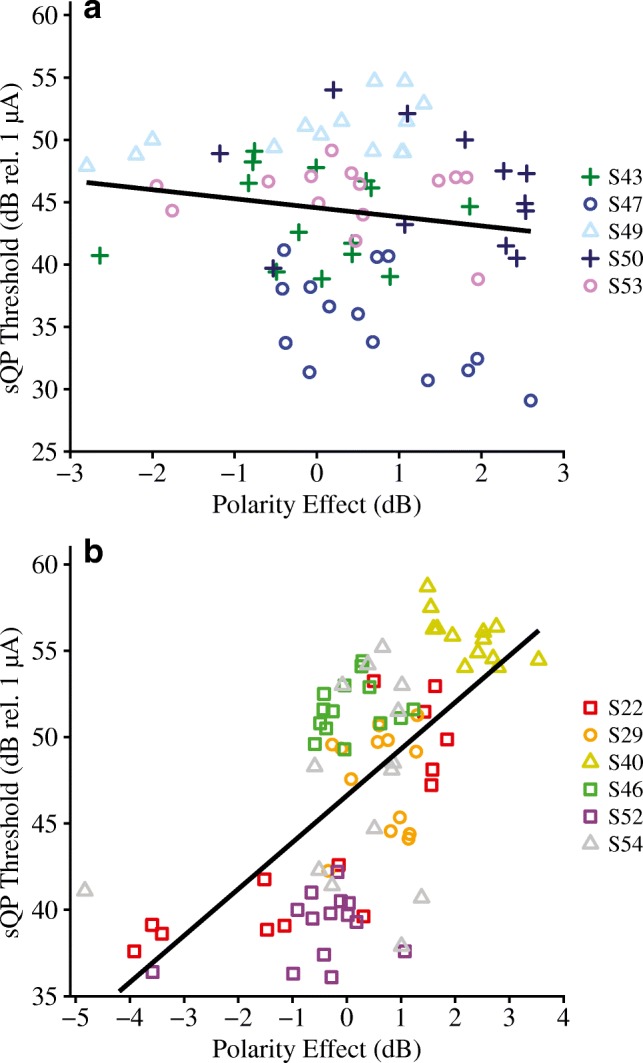
Focused (sQP) thresholds (in dB relative to 1 μA) as a function of polarity effect (in dB) for two groups of subjects that are grouped based on the strength of their within-subject correlations between electrode-to-modiolus distance (in mm) and sQP thresholds. **a** Subjects that have a very strong correlation (*r* = 0.71 to 0.94) between electrode-to-modiolus distance and sQP thresholds. **b** Subjects that have a weak-to-moderate correlation (*r* = 0.06 to 0.48) between electrode-to-modiolus distance and sQP thresholds. Individual subjects are represented by different colors and shapes

Subjects were separated into two groups based on their threshold-EMD correlations for further analysis. Subjects with threshold-EMD correlation coefficients larger than 0.70 were in the “strong threshold-EMD” group (*N* = 5), whereas those below the 0.70 cutoff were in the “weak-to-moderate threshold-EMD” group (*N* = 6). Figure 6 shows the relationship between the polarity effect and sQP thresholds for (a) the five subjects with strong and statistically significant threshold-EMD correlations, and (b) the six subjects with weak-to-moderate, non-significant threshold-EMD correlations. Repeated measures correlations were performed to determine the strength of the relationship between sQP thresholds and the polarity effect within each group. Overall, the group with strong threshold-EMD correlations did not demonstrate a significant relationship between sQP thresholds and the polarity effect (*r*_rm(63)_ = 0.03, 95 % CI [− 0.22, 0.28], *P* = 0.80). However, the relationship between the polarity effect and sQP thresholds was significant for the group with weak-to-moderate threshold-EMD correlations (*r*_rm(76)_ = 0.48, 95 % CI [0.28, 0.63], *P* < 0.001). These results emphasize that sQP thresholds can reflect either electrode position or neural integrity (as estimated by the polarity effect). Relationships between sQP thresholds and neural integrity may be more likely to emerge when the variation in sQP thresholds cannot be explained by variation in electrode position.

### Polarity Sensitivity and Duration of Deafness

**Fig. 7:**
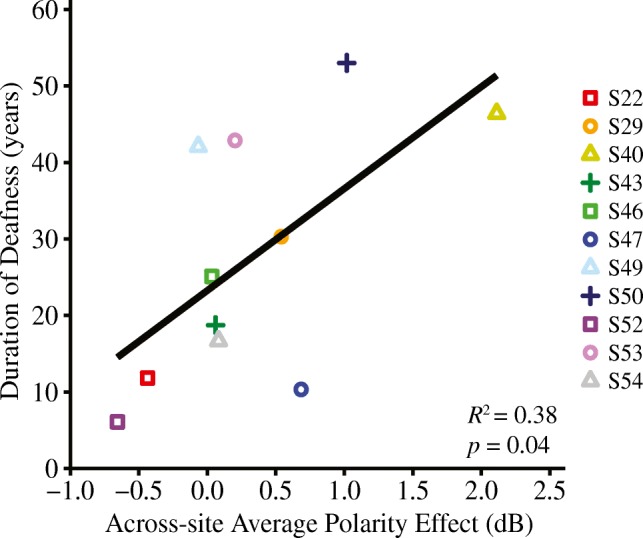
The relationship between duration of deafness (in years) and across-site average polarity effect (in dB) is shown (*R*^2^ = 0.38, *P* = 0.04). Individual subjects are represented by different colors and shapes

Finally, we performed a preliminary analysis to quantify the relationship between the polarity effect and duration of deafness, a common implicit assessment of neural integrity. Figure 7 shows the relationship between duration of deafness and the across-site average polarity effect. For this analysis, the polarity effect was averaged across all available electrodes for each subject. Duration of deafness was defined as the time, in years, between diagnosis of severe-to-profound sensorineural hearing loss and CI activation. A linear regression analysis indicated that duration of deafness was significantly correlated with the across-site average polarity effect (*R*^2^ = 0.38, *R*^2^_adjusted_ = 0.32, *F*_(1,9)_ = 5.62, *P* = 0.04). Specifically, individuals that experienced relatively long durations of deafness prior to receiving a CI also tended to have relatively large, positive polarity effects.

## DISCUSSION

The present study evaluated the theory that polarity sensitivity reflects neural status in CI listeners. An ideal estimate of neural integrity should vary independently of other factors that influence the quality of the CI electrode-neuron interface, such as electrode position and tissue impedances. Our primary results support these tenets; specifically, the polarity effect at threshold was subject- and electrode-dependent and varied independently of CT-estimated electrode position and intracochlear resistance. The polarity effect was also positively correlated with focused behavioral thresholds, which are believed to reflect a combination of local neural status, electrode position, and intracochlear bone and tissue growth. Importantly, the polarity effect remained a significant predictor of focused thresholds after statistically controlling for electrode position and intracochlear resistance. Taken together, these results support the theory that polarity sensitivity may reflect neural health in CI listeners.

### Polarity Sensitivity Varies Independently of Electrode Position and Intracochlear Resistance

Psychophysical polarity sensitivity at threshold varied across- and within-subjects. These findings are consistent with previous investigations that demonstrated subject- and electrode-dependent polarity effects at threshold (Macherey et al. 2017; Carlyon et al. 2018). However, previous reports did not rule out the possibility that channel-to-channel variability in the polarity effect was influenced by non-physiological factors. It is possible that either the physical distance of an electrode to its target neurons or the location of the electrode relative to the central axons could influence polarity sensitivity measurements.

The results of the present study demonstrate that the polarity effect at threshold is independent of CT-estimated electrode-to-modiolus distance and electrode scalar location. These findings may be explained by the nature of the polarity effect measurement, which reflects a difference score rather than an absolute threshold response. Theoretically, excitability (i.e., absolute threshold) of degenerated neurons depends on the physical distance between the electrode and the soma (Rattay et al. 2001b). At a single electrode site, the effect of physical distance on absolute threshold response to each polarity should cancel out when a difference score (i.e., polarity effect) is calculated.

Moreover, recall that modeling work suggests that anodic and cathodic polarities have different sites of spike initiation, wherein depolarization occurs near the central axon for anodic stimuli and more peripherally for cathodic stimuli (Rattay et al. 2001a, b; Joshi et al. 2017; Resnick et al. 2018). When the peripheral processes have degenerated, depolarization in response to cathodic current would occur near the unmyelinated cell body, which is difficult to excite in humans (Rattay 1999; Rattay et al. 2001a). Conversely, for anodic stimulation, action potentials are expected to be generated near the central axon, and therefore do not need to overcome the unmyelinated soma. In turn, when the peripheral processes have degenerated, thresholds in response to cathodic stimuli will be higher than those in response to anodic stimuli (i.e., positive polarity effect).

Conceivably, then, electrode position relative to the central axon could influence polarity sensitivity. However, basic principles of human cochlear anatomy and CI electrode placement would suggest that a functioning CI electrode must be located within the cochlear compartments, distal/peripheral to the soma. In humans, SGN somata are located within Rosenthal’s canal; each cell body extends a peripheral process toward the organ of Corti and a central process into the auditory nerve (Nayagam et al. 2011). Since it is unlikely that a viable CI electrode would be located proximal/central to the soma, the difference in threshold response to anodic versus cathodic stimulation should be independent of the position of the electrode relative to the modiolus.

In addition to electrode position, intracochlear bone and fibrous tissue growth are believed to vary within- and across-subjects (Spelman et al. 1982; Bierer et al. 2015a; Kamakura and Nadol 2016). The present results showed that the polarity effect varies independently of EFI-estimated longitudinal intracochlear resistance. Again, these findings are consistent with the polarity effect measurement as a difference score between threshold responses to two stimuli. Presumably, responses to anodic and cathodic polarities would be affected in similar ways by the impedance environment near a recording electrode; thus, effects of intracochlear resistance on threshold measurements should cancel out when calculating the difference between responses to each polarity at a given site.

In summary, the polarity effect at threshold varies independently of electrode position relative to the modiolus, electrode scalar location, and intracochlear resistance. This may be related to the nature of the polarity effect measurement (i.e., difference score rather than absolute threshold response) and to the necessary position of functioning CI electrodes in humans (i.e., somewhat distal to the SGN cell bodies).

### Across Subjects, the Polarity Effect Is Related to Focused Behavioral Thresholds

Several lines of evidence suggest that focused behavioral thresholds are related to electrode position within the cochlea, intracochlear resistance, and estimates of neural integrity (e.g., Bierer 2007; Bierer and Faulkner 2010; Goldwyn et al. 2010; Bierer et al. 2011; Bierer and Nye 2014; Bierer et al. 2015b; Long et al. 2014; DeVries et al. 2016). Thus, if polarity sensitivity reflects peripheral degeneration in CI listeners, then it should explain some of the variation in focused behavioral thresholds, even after accounting for electrode position and intracochlear resistance. The present results confirmed this hypothesis.

Specifically, electrodes with relatively high focused behavioral thresholds also tended to have relatively large, positive polarity effects. This relationship held after statistically controlling for CT-estimated electrode position and EFI-estimated intracochlear resistance, which are known to partially explain variation in focused thresholds (Long et al. 2014; Bierer et al. 2015a; DeVries et al. 2016; DeVries and Arenberg 2018a). These findings agree with modeling data showing that electrodes near regions with degenerated or demyelinated neurons have higher thresholds than electrodes near regions with healthy spiral ganglion populations (Goldwyn et al. 2010; Joshi et al. 2017; Resnick et al. 2018). The present data are also consistent with Carlyon et al. (2018), who found a modest, but significant, correlation between the polarity effect and the average of ACA and CAC monopolar thresholds in a sample of eight adults with CIs. Taken together, these findings provide support for the theory that polarity sensitivity may reflect neural health in CI listeners.

Consistent with previous investigations, we also observed a strong relationship between CT-estimated electrode position and focused thresholds (Long et al. 2014; DeVries et al. 2016; DeVries and Arenberg 2018a). Specifically, electrodes that were located far from their target neurons, or that were translocated to SV, had relatively high focused thresholds. We further demonstrated that lower intracochlear resistance values are predictive of higher focused thresholds. The negative relationship between focused thresholds and intracochlear resistance can be explained in the context of Ohm’s Law, wherein the amount of current necessary to achieve a criterion voltage (i.e., threshold level) is inversely related to the resistance in the system. These results are consistent with those of Bierer et al. (2015b), who demonstrated that lower monopolar and tripolar thresholds are associated with higher impedance values.

However, despite the overall relationship between the polarity effect and focused thresholds, Fig. 5 b and Table 3 show that positive and significant within-subject correlations were not always observed. Examining the threshold-PE correlations in the context of the individual correlations between focused thresholds and electrode-to-modiolus distance (threshold-EMD) revealed a possible explanation for this finding. Five subjects had strong and significant threshold-EMD correlations (ranging from *r* = 0.71 to 0.94). In those individuals, the variation in focused thresholds was largely explained by variation in electrode position. In fact, the across-subject correlation between the polarity effect and focused thresholds was driven by individuals with weak or moderate threshold-EMD relationships (Fig. 6). This observation emphasizes the importance of developing in vivo neural health measures that vary independently of confounding factors such as electrode position. In isolation, behavioral thresholds cannot provide a reliable indication of the integrity of local SGNs. Furthermore, it is likely that an ideal assessment of a CI listener’s electrode-neuron interface involves a comprehensive evaluation of both electrode position and neural health.

Of note, it is possible that correlations between the polarity effect and focused thresholds could improve with the use of equivalent stimulation rates to measure each response. Stimulation rate is known to influence psychophysical thresholds, wherein thresholds tend to decrease with increasing pulse rate (e.g., Pfingst et al. 2011; Zhou et al. 2012). For the present study, the stimuli used to measure focused thresholds and the polarity effect were selected in keeping with previous literature. Moreover, given that the polarity effect was represented by a difference score, the effect of stimulation rate on absolute thresholds is not expected to substantially contribute to the outcome measure. However, potential interactions between stimulation rate and polarity effect measurements remain to be investigated.

### Clinical Implications and Future Directions

Overall, the present results support the theory that the psychophysical polarity effect at threshold may reflect local neural health in CI listeners. Results also indicated that individuals with relatively long durations of deafness prior to implantation tend to have relatively large, positive polarity effects. Although duration of deafness is merely an implicit correlate of neural health, this finding aligns with temporal bone analyses showing that low total SGN counts are associated with long periods of auditory deprivation (Nadol et al. 1989).

Individual- and electrode-specific differences in the magnitude of the polarity effect suggest that this measurement has the potential to assist in developing individualized programming recommendations. However, if polarity sensitivity reflects local neural health in CI listeners, it remains to be determined what magnitude of polarity effect is meaningful. It may be that only extreme positive or negative values provide useful insight into the health of local neurons. In fact, Resnick et al. (2018) predicted that only extreme demyelination results in pathological current spread and recruitment. Future studies with larger samples should investigate the cumulative contributions of diverse aspects of peripheral health to auditory perception with a CI.

Ideally, a combination of neural health and electrode position estimates would be used to optimize the electrode-neuron interface for an individual subject. DeVries and Arenberg (2018b) found that implementing current focusing on electrodes that are located far from the modiolus may help to reduce channel interaction. Some CI listeners also experience speech perception benefit when electrodes that are estimated to interface poorly with the auditory nerve are deactivated (Noble et al. 2015; Bierer and Litvak 2016; Zhou 2017). Together, assessments of neural health and electrode position could be used to comprehensively characterize neural function near each electrode site, ultimately leading to improved patient-specific interventions. Future work should determine potential uses of polarity sensitivity in individualized CI programming adjustments.

## References

[CR1] Bakdash JZ, Marusich LR (2018) rmcorr: Repeated measures correlation. R package version 0.3.0. https://CRAN.R-project.org/package=rmcorr

[CR2] Bakdash JZ, Marusich LR (2017). Repeated measures correlation. Front Psychol.

[CR3] Bartón K (2018) MuMIn: multi-model inference. R package version 1.42.1. Retrieved August 15, 2018, from http://cran.r-project.org/package=MuMIn

[CR4] Bates D, Maechler M, Bolker B, Walker S (2015). Fitting linear mixed-effects models using lme4. J Stat Softw.

[CR5] Bierer JA (2007). Threshold and channel interaction in cochlear implant users: evaluation of the tripolar electrode configuration. J Acoust Soc Am.

[CR6] Bierer JA (2010). Probing the electrode-neuron interface with focused cochlear implant stimulation. Trends Amplif.

[CR7] Bierer JA, Faulkner KF (2010). Identifying cochlear implant channels with poor electrode-neuron interface: partial tripolar, single-channel thresholds and psychophysical tuning curves. Ear Hear.

[CR8] Bierer JA, Litvak L (2016). Reducing channel interaction through cochlear implant programming may improve speech perception: current focusing and channel deactivation. Trends Hear.

[CR9] Bierer JA, Nye AD (2014). Comparisons between detection threshold and loudness perception for individual cochlear implant channels. Ear Hear.

[CR10] Bierer JA, Faulkner KF, Tremblay KL (2011). Identifying cochlear implant channels with poor electrode-neuron interfaces: electrically evoked auditory brain stem responses measured with the partial tripolar configuration. Ear Hear.

[CR11] Bierer JA, Bierer SM, Kreft HA, Oxenham AJ (2015). A fast method for measuring psychophysical thresholds across the cochlear implant array. Trends Hear.

[CR12] Bierer SM, Shea-Brown E, Bierer JA (2015b) Current spread in the cochlea: insights from CT and electrical field imaging. Poster presented at the Conference on Implantable Auditory Prostheses, Tahoe, CA

[CR13] Briare JJ, Frijns JHM (2000). Field patterns in a 3D tapered spiral model of the electrically stimulated cochlea. Hear Res.

[CR14] Carlyon RP, Deeks JM, Macherey O (2013). Polarity effects on place pitch and loudness for three cochlear-implant designs and at different cochlear sites. J Acoust Soc Am.

[CR15] Carlyon RP, Cosentino S, Deeks JM, Parkinson W, Arenberg JG (2018). Effect of stimulus polarity on detection thresholds in cochlear implant users: relationships with average threshold, gap detection, and rate discrimination. J Assoc Res Otolaryngol.

[CR16] Cohen J (1988). Set correlation and contingency tables. Appl Psychol Meas.

[CR17] DeVries L, Arenberg JG (2018). Psychophysical tuning curves as a correlate of electrode position in cochlear implant listeners. J Assoc Res Otolaryngol.

[CR18] DeVries L, Arenberg JG (2018). Current focusing to reduce channel interaction for distant electrodes in cochlear implant programs. Trends Hear.

[CR19] DeVries L, Scheperle R, Bierer JA (2016). Assessing the electrode-neuron interface with the electrically-evoked compound action potential, electrode position, and behavioral thresholds. J Assoc Res Otolaryngol.

[CR20] Dhanasingh A, Jolly C (2017). An overview of cochlear implant electrode array designs. Hear Res.

[CR21] Dietz A, Wennström M, Lehtimäki A, Löppönen H, Valtonen H (2016). Electrode migration after cochlear implant surgery: more common than expected?. Eur Arch Otorhinolaryngol.

[CR22] Friedland DR, Runge-Samuelson C, Baig H, Jensen J (2010). Case-control analysis of cochlear implant performance in elderly patients. Arch Otolaryngol Head Neck Surg.

[CR23] Goldwyn JH, Bierer SA, Bierer JA (2010). Modeling the electrode-neuron interface of cochlear implants: effects of neural survival, electrode placement, and the partial tripolar configuration. Hear Res.

[CR24] Holden LK, Finley CC, Firzst JB, Holden TA, Brenner C, Potts LG, Gotter BD, Vanderhoof SS, Mispagel K, Heydebrand G, Skinner MW (2013). Factors affecting open-set word recognition in adults with cochlear implants. Ear Hear.

[CR25] Hughes ML (2012) Objective measures in cochlear implants. http://ebookcentral.proquest.com. Accessed 15 Jan 2019

[CR26] Hughes ML, Goehring JL, Baudhuin JL (2017). Effects of stimulus polarity and artifact reduction method on the electrically evoked compound action potential. Ear Hear.

[CR27] Hughes ML, Sangsook C, Glickman E (2018). What can stimulus polarity and interphase gap tell us about auditory nerve function in cochlear-implant recipients?. Hear Res.

[CR28] Hurvich CM, Tsai C (1989). Regression and time series model selection in small samples. Biometrika.

[CR29] Jolly CN, Spelman FA, Clopton BM (1996). Quadrupolar stimulation for cochlear prostheses: modeling and experimental data. IEEE Trans Biomed Eng.

[CR30] Joshi SN, Dau T, Epp B (2017). A model of electrically stimulated auditory nerve fiber responses with peripheral and central sites of spike generation. J Assoc Res Otolaryngol.

[CR31] Kamakura A, Nadol JB (2016). Correlation between word recognition score and intracochlear new bone and fibrous tissue after cochlear implantation in the human. Hear Res.

[CR32] Kim J-R, Abbas PJ, Brown CJ, Etler CP, O’Brien S, Kim L-S (2010). The relationship between electrically evoked compound action potential and speech perception: a study in cochlear implant users with short electrode array. Otol Neurotol.

[CR33] Kuznetsova A, Brokhoff PB, Christensen RHB (2017). lmerTest package: tests in linear mixed effects models. J Stat Softw.

[CR34] Lazard DS, Vincent C, Venail F, Van de Heyning P, Truy E, Sterkers O, Skarzynski PH, Skarzynski H, Schauwers L, O’Leary S, Mawman D, Maat B, Kleine-Punte A, Huber AM, Green K, Govaerts PJ, Fraysse B, Dowell R, Dillier N, Burke E, Beynon A, Bergeron F, Baskent D, Artieres F, Blamey PJ (2012). Pre-, per- and postoperative factors affecting performance of postlinguistically deaf adults using cochlear implants: a new conceptual model over time. PLoS One.

[CR35] Long CJ, Holden TA, McClelland GH, Parkinson WS, Shelton C, Kelsall DC, Smith ZM (2014). Examining the electro-neural interface of cochlear implant users using psychophysics, CT scans, and speech understanding. J Assoc Res Otolaryngol.

[CR36] Macherey O, van Wieringen A, Carlyon RP, Deeks JM, Wouters J (2006). Asymmetric pulses in cochlear implants: effects of pulse shape, polarity, and rate. J Assoc Res Otolaryngol.

[CR37] Macherey O, Carlyon RP, van Wieringen A, Deeks JM, Wouters J (2008). Higher sensitivity of human auditory nerve fibers to positive electrical currents. J Assoc Res Otolaryngol.

[CR38] Macherey O, Carlyon RP, Chatron J, Roman S (2017). Effect of pulse polarity on thresholds and on non-monotonic loudness growth in cochlear implant users. J Assoc Res Otolaryngol.

[CR39] McNeish D (2017). Small sample methods for multilevel modeling: a colloquial elucidation of REML and the Kenward-Roger correction. Multivar Behav Res.

[CR40] Nadol JB (1997). Patterns of neural degeneration in the human cochlea and auditory nerve: implications for cochlear implantation. Otolaryngol Head Neck Surg.

[CR41] Nadol JB, Young YS, Glynn RJ (1989). Survival of spiral ganglion cells in profound sensorineural hearing loss: implications for cochlear implantation. Ann Otol Rhinol Laryngol.

[CR42] Nakagawa S, Schielzeth H (2013). A general and simple method for obtaining *R*^2^ from generalized linear mixed-effects models. Methods Ecol Evol.

[CR43] Nayagam BA, Muniak MA, Ryugo DK (2011). The spiral ganglion: connecting the peripheral and central auditory systems. Hear Res.

[CR44] Noble JH, Gifford RH, Hedley-Williams AJ, Dawant BM, Labadie RF (2015). Clinical evaluation of an image-guided cochlear implant programming strategy. Audiol Neurootol.

[CR45] Pfingst BE, Colesa DJ, Hembrador S, Kang SY, Middlebrooks JC, Raphael Y, Su GL (2011). Detection of pulse trains in the electrically stimulated cochlea: effects of cochlear health. J Acoust Soc Am.

[CR46] R Core Team (2016) R: a language and environment for statistical computing. R Foundation for Statistical Computing. https://www.R-project.org/

[CR47] Rader T, Baumann U, Stöver T, Weissgerber T, Adel Y, Leinung M, Helbig S (2016). Management of cochlear implant electrode migration. Otol Neurotol.

[CR48] Rattay F (1999) The basic mechanism for the electrical stimulation of the nervous system. Neuroscience 89(2):335–346 10.1016/s0306-4522(98)00330-3 10077317

[CR49] Rattay F, Lutter P, Felix H (2001). A model of the electrically excited human cochlear neuron I. Contribution of neural substructures to the generation and propagation of spikes. Hear Res.

[CR50] Rattay F, Lutter P, Felix H (2001). A model of the electrically excited human cochlear neuron II. Influence of the three-dimensional cochlear structure on neural excitability. Hear Res.

[CR51] Resnick JM, O’Brien GE, Rubinstein JT (2018). Simulated auditory nerve axon demyelination alters sensitivity and response timing to extracellular stimulation. Hear Res.

[CR52] Robb RA (2001). The biomedical imaging resource at Mayo Clinic. IEEE Trans Med Imaging.

[CR53] Scheperle RA (2017). Suprathreshold compound action potential amplitude as a measure of auditory function in cochlear implant users. J Otol.

[CR54] Schvartz-Leyzac KC, Pfingst PE (2018). Assessing the relationship between the electrically evoked compound action potential and speech recognition abilities in bilateral cochlear implant recipients. Ear Hear.

[CR55] Sek A, Alcantara J, Moore BCJ, Kluk K, Wicher A (2005). Development of a fast method for determining psychophysical tuning curves. Int J Audiol.

[CR56] Skinner MW, Holden TA, Whiting BR, Voie AH, Brunsden B, Neely G, Saxon EA, Hullar TE, Finley CC (2007). In vivo estimates of the position of Advanced Bionics electrode arrays in the human cochlea. Ann Otol Rhinol Laryngol.

[CR57] Spelman FA, Clopton BM, Pfingst BE (1982). Tissue impedance and current flow in the implanted ear. Implications for the cochlear prosthesis. Ann Otol Rhinol Laryngol Suppl.

[CR58] Teymouri J, Hullar TE, Holden TA, Chole RA (2011). Verification of computed tomographic estimates of cochlear implant array position: a micro-CT and histologic analysis. Otol Neurotol.

[CR59] Undurraga JA, van Wieringen A, Carlyon RP, Macherey O, Wouters J (2010). Polarity effects on neural responses of the electrically stimulated auditory nerve at different cochlear sites. Hear Res.

[CR60] Undurraga JA, Carlyon RP, Wouters J, van Wieringen A (2013). The polarity sensitivity of the electrically stimulated human auditory nerve measured at the level of the brainstem. J Assoc Res Otolaryngol.

[CR61] van Wieringen A, Macherey O, Carlyon RP, Deeks JM, Wouters J (2008). Alternative pulse shapes in electrical hearing. Hear Res.

[CR62] Vanpoucke FJ, Zarowski AJ, Peeters SA (2004). Identification of the impedance model of an implanted cochlear prosthesis from intracochlear potential measurements. IEEE Trans Biomed Eng.

[CR63] Voie AH, Burns DH, Spelman FA (1993). Orthogonal-plane fluorescence optical sectioning: three-dimensional imaging of macroscopic biological specimens. J Microsc.

[CR64] Zhou N (2017). Deactivating stimulation sites based on low-rate thresholds improves spectral ripple and speech reception thresholds in cochlear implant users. J Acoust Soc Am.

[CR65] Zhou N, Pfingst BE (2014). Relationship between multipulse integration and speech recognition with cochlear implants. J Acoust Soc Am.

[CR66] Zhou N, Xu L, Pfingst BE (2012). Characteristics of detection thresholds and maximum comfortable loudness levels as a function of pulse rate in human cochlear implant users. Hear Res.

